# The relationship between morphological awareness and word reading in Brazilian Portuguese: a longitudinal study

**DOI:** 10.1186/s41155-022-00245-9

**Published:** 2023-02-03

**Authors:** Francis Ricardo dos Reis Justi, Bruno Stefani Ferreira de Oliveira, Cláudia Nascimento Guaraldo Justi

**Affiliations:** Department of Psychology, Federal University of Juiz de Fora, Juiz de Fora, MG 36036-330 Brazil

**Keywords:** Morphological awareness, Reading, Phonological working memory, Phonological awareness

## Abstract

Studies have provided evidence that morphological awareness contributes to word reading in opaque languages such as English. However, this relationship is not yet established for Brazilian Portuguese, a much less opaque language. The present study performed a longitudinal investigation of the relationship between morphological awareness and word reading in 162 children from 2nd to 5th grades of elementary school. The children were evaluated in the final quarter of the respective school year (time 1) and 1 year later (time 2). Hierarchical regression analyses controlling for intelligence, phonological awareness, and phonological working memory were conducted. The hierarchical regression analyses were followed up by cross-lagged panel correlations, and both results converged to word reading measured in the 2nd and 3rd grades contributing to morphological awareness 1 year later. In addition, morphological awareness measured in the 4th grade contributed to word reading 1 year later. The greater transparency of Brazilian Portuguese may make morphological awareness less important for word reading in the early grades; however, in older children, morphological awareness is important for word reading performance.

**Supplementary Information:**

The online version contains supplementary material available at 10.1186/s41155-022-00245-9.

Alphabetical linguistic systems exhibit a strong relationship between letters and speech sounds. Thus, several studies have shown that phonological awareness, the ability to manipulate and reflect on speech sounds, has a positive and significant relationship with the ability to read and write (see Melby-Lervåg et al., 2012 for a review). However, reading is more than converting letters into sounds, and for comprehension to occur, phonological forms need to be mapped onto semantic information (Kuo & Anderson, 2006). Morphemes are defined as the smallest units of meaning, and a great deal of words can be analyzed into these smaller meaningful parts such as prefixes, roots, and suffixes (Nagy et al., 2014). Thus, morphological awareness, the ability to reflect on and to manipulate the morphemic structure of words (Carlisle, 1995), could play a role in learning to read and write. Several studies in different languages that control for phonological awareness and intelligence show a significant relationship between scores on tasks that measure morphological awareness and scores on tasks that measure reading comprehension (e.g., Carlisle, 1995; Deacon et al., 2018; Kirby et al., 2012; Kruk & Bergman, 2013; Levesque et al., 2018; Lyster et al., 2021; Nagy et al., 2006; Oliveira et al., 2020; Pittas & Nunes, 2014; Ruan et al., 2018; To et al., 2016).

There are also studies about the relationship between morphological awareness and single word reading; however, the results of these studies are mixed, with morphological awareness contributing to word reading in some studies (e.g., Deacon et al., 2013; Diamanti et al., 2017; Kruk & Bergman, 2013; Muroya et al., 2017) but not in others (e.g., Colé et al., 2018; Deacon & Kirby, 2004; Law & Ghesquière, 2017; Manolitsis et al., 2017; Simpson et al., 2020). For example, in English, Deacon and Kirby (2004) conducted a 4-year (grades 2 to 5) longitudinal study. In this study, after controlling for intelligence and phonological awareness, morphological awareness did not contributed for single word reading (although it contributed for pseudoword reading in the 4th and 5th grades). On the other hand, Deacon et al. (2013) observed that morphological awareness assessed in the 2nd grade contributed to a composite measure of reading (words and pseudowords) in the 3rd grade. The same kind of mixed results appears to happen in other languages like Greek. For example, after controlling for vocabulary and phonological awareness, Diamanti et al. (2017) have shown that morphological awareness assessed in the kindergarten predicted word reading accuracy in grade 1. On the other hand, in a study that controlled for vocabulary, nonverbal IQ, phonological awareness, and rapid automatized naming, Manolitsis et al. (2017) have not detected a contribution of morphological awareness assessed in grade 1 to word reading fluency assessed both in the end of grade 1 and in the second grade.

Complicating matters even further, Desrochers et al. (2018) have demonstrated that language can be a modulating factor on the relationship between morphological awareness and reading. In their study, Desrochers et al. (2018) compared children of different countries learning to read in English, French, or Greek. The three writing systems differ greatly in their degree of consistency in grapheme-phoneme mapping with English being the most inconsistent, followed by French and Greek (representing a system with a very consistent grapheme-phoneme mapping). Desrochers et al. (2018) observed that morphological awareness predicted single word reading in 2nd graders learning to read in English (reading accuracy and fluency) and French (reading fluency), but not in 2nd graders learning to read in Greek. Thus, it seems that in inconsistent writing systems such as English and French, morphological awareness predicts word reading very early. However, it is important to notice that morphological awareness predicted reading comprehension in the three writing systems.

In another recent study, Mousikou et al. (2020) suggest that language could modulate morphological processing during reading aloud. In their study, Mousikou et al. compared children learning to read in English, French, German, and Italian. Children from the 3rd grade performed a reading aloud task with words and pseudowords. The critical manipulation was in the pseudowords which belonged to four conditions: stem + suffix, stem + non-suffix, non-stem + suffix, and non-stem + non-suffix. They observed a main effect of stem and suffix, that is, pseudowords made of real stem and real suffix were named faster. However, they observed interactions between stem and language and between suffix and language. These interactions occurred mainly because the stem and suffix effects were much larger in English than in the other languages, suggesting that developing readers of English use morphology in reading aloud to a greater extent than readers of French, German, and Italian. A similar pattern of results was observed in adult proficient readers in these four languages. Thus, morphological processing appears to be more robust in English than in more consistent orthographies. It is important to notice that the kind of tacit morphological processing measured by Mousikou et al. is different from the strategic and conscious application of morphological knowledge as measured by Desrochers et al. (2018) morphological awareness’ tasks. However, as Nagy et al. (2014) point out, this limit is not easy to set, and measures of morphological awareness necessarily draw on both, reflecting differences in the awareness of morphological relationships between words and in the tacit morphological processing of words.

The studies by Desrochers et al. (2018) and Mousikou et al. (2020) suggest that the results observed in the English language do not necessarily apply in the same way to other languages. This state of affairs highlights the importance of conducting reading research in other languages besides English (Share, 2008). Unfortunately, when we consider longitudinal studies designed to investigate the direction of the relationship between morphological awareness and single word reading, the number of studies in other languages besides Chinese and English is limited. In an attempt to reduce this gap in the literature, the present study reports a longitudinal investigation of the relationship between morphological awareness and single word reading in Brazilian Portuguese. A second important question investigated in the present study is whether the contribution of morphological awareness to reading ability changes with development (e.g., Kirby et al., 2012; Kuo & Anderson, 2006). For example, in Kirby et al. (2012) study, after controlling for IQ and phonological awareness, morphological awareness measured in the 1st grade did not contribute to word reading in the 3rd grade (neither accuracy nor speed). Although when morphological awareness was measured in the 2nd grade, it contributed to word reading accuracy, and when measured in the 3rd grade, it contributed to both word reading accuracy and speed. Thus, it is possible to argue that the relationship between morphological awareness and reading gets stronger as children advance through grades, possibly because the texts they are expected to read contain more morphologically complex words (Kuo & Anderson, 2006). To address this question, we followed children from the 2nd, 3rd, 4th, and 5th grades for 1 year. Since nonverbal intelligence, phonological awareness, and phonological working memory are well-established predictors of reading that share variance with morphological awareness (e.g., Deacon & Kirby, 2004; Justi & Roazzi, 2012), we included controls for these variables in the present study. In the following, we characterize Brazilian Portuguese and review the literature concerning morphological awareness and reading in this language. After this, we discuss some theoretical accounts on how morphological awareness relates to single word reading and present the rationale for the present study hypotheses.

## Brazilian Portuguese

Portuguese is an inflectional Romance language spoken in countries of four continents: Africa, Asia, Europe, and South America (Estivalet & Meunier, 2015). In comparison with the complex syllabic structure of Germanic languages, Portuguese has a simple syllabic structure with a reasonable number of open CV (consonant–vowel) syllables (Seymour, 2005). On the other hand, considering its grapheme-phoneme mapping, some researches characterize Portuguese as being in an intermediate position between deep orthographies, such as English (which has the less consistent grapheme-phoneme mapping), and shallow orthographies, such as Finnish and Italian, which have very consistent grapheme-phoneme mappings (Lima e Castro, 2010; Ziegler et al., 2010; Seymour et al., 2003). However, since different criteria can be employed, it is not an easy task to classify an orthography as shallow or deep (Schmalz et al., 2015). According to Schmalz et al. (2015), one can consider the complexity of the letter-to-sound mapping which can be simple one letter to one phoneme, or complex, involving multiple letters to one phoneme. Considering this dimension, Brazilian Portuguese is relatively simple with a good deal of one letter to one phoneme mappings and a set of simple contextual rules governing the pronunciation of some letters. For example, the letter < s > between vowels is mapped to the phoneme /z/.

Another dimension discussed by Schmalz et al. (2015) is the consistency of the grapheme-phoneme mapping, that is, to what extent this mapping is always the same. In Brazilian Portuguese, most words either are completely consistent in their grapheme-phoneme mapping, or they can be read aloud correctly with a few contextual rules (Pinheiro, Lucio & Silva, 2008). According to Pinheiro et al. (2008), there are three possibilities for inconsistent grapheme-phoneme mapping in Brazilian Portuguese: (1) the grapheme < x > which can represent the phonemes /s/ (e.g., “texto” [TEXT]), /z/ (e.g., “exato” [EXACT]), /ks/ (e.g., “boxe” [BOXING]), and /ʆ/ (e.g., “lixo” [TRASH]); (2) the grapheme < e > which can represent the phonemes /e/ (e.g., “pelo” [FUR]) and /Ɛ/ (e.g., “meta” [GOAL]); and (3) the grapheme < o > which can represent the phonemes /o/ (e.g., “soco” [PUNCH]) and /Ͻ/ (e.g., “foca” [SEAL]). In the case of the graphemes < e > and < o > , the inconsistency occurs only when they are in the stressed syllable of trochaic/paroxytone words, i.e., they are the vowel of stressed syllable followed by an unstressed last syllable.

Another way to index grapheme-phoneme ambiguity is in terms of entropy (Borgwaldt et al., 2005). In this approach, if a given grapheme always corresponds to one specific phoneme, the mapping is completely predictable, and the corresponding entropy value is zero; as far as this mapping becomes less predictable, the higher its entropy value will be (Borleffs et al., 2017). Borgwaldt et al. (2005) calculate word-initial letter-phoneme entropies for seven languages: Dutch, English, French, German, Hungarian, Italian, and Brazilian Portuguese. In their analysis, Brazilian Portuguese was ranked in the middle of the orthographic deep spectrum close to French and German, while English represented the less predictable orthography and Hungarian and Italian the most predictable. Overall, these results are in accordance with other studies (Lima e Castro, 2010; Ziegler et al., 2010; Seymour et al., 2003) classifying Brazilian Portuguese in the middle of the orthographic deep spectrum.

## Morphological awareness and reading in Brazilian Portuguese

In Brazil, reading instruction begins in the first grade of elementary school (although in an unsystematic way) with children around 6 to 7 years old, and reading is supposed to be fully automatized only at the end of the 3rd grade. According to Sargiani et al. (2018), literacy instruction in Brazil is mainly based on a meaning-centered approach in which grapheme-phoneme correspondences are only incidentally taught. Considering the relationship between morphological awareness and spelling in Brazilian Portuguese, there is consistent evidence for an independent contribution of morphological awareness to spelling (e.g., Mota, Anibal & Lima, 2008; Mota & Silva, 2007; Justi & Roazzi, 2012). In addition, there is solid evidence for an independent contribution of morphological awareness to reading comprehension, even after controlling for phonological awareness, IQ, or vocabulary (e.g., De Freitas et al., 2018; Mota et al., 2012). For example, Mota et al. (2012) investigated longitudinally the relationship between reading comprehension and morphological awareness in second- and third-grade Brazilian children. In this study, even after controlling for school grade, vocabulary, and phonological awareness, morphological awareness predicted reading comprehension 1 year later.

On the other hand, similarly to studies in other languages (e.g., Colé et al., 2018; Deacon & Kirby, 2004; Deacon et al., 2013; Diamanti et al., 2017; Kruk & Bergman, 2013; Law & Ghesquière, 2017; Manolitsis et al., 2017; Muroya et al., 2017; Simpson et al., 2020), the relationship between morphological awareness and single word reading in Brazilian Portuguese is not clear. An important issue in this situation is if morphological awareness can contribute to word reading in Brazilian Portuguese after the statistical control of phonological awareness. However, the results of the studies which applied this kind of statistical control are mixed; some studies have found an independent contribution of morphological awareness to word reading (e.g., De Freitas et al., 2018; Oliveira & Justi, 2017a), whereas others have not (e.g., Justi & Roazzi, 2012; Miranda & Mota, 2013). In de Freitas et al. (2018) study, morphological awareness predicted word reading (accuracy and fluency) and reading comprehension in 4th graders, even after controlling for nonverbal intelligence and phonological awareness. On the other hand, in a cross-sectional study, Oliveira and Justi (2017a) did not find a statistically significant effect of morphological awareness for word reading accuracy in 2nd and 3rd graders after controlling for nonverbal intelligence, phonological awareness, and phonological working memory. However, in 4th and 5th graders, morphological awareness had a statistically significant effect on word reading accuracy, even after controlling for nonverbal intelligence, phonological awareness, and phonological working memory.

Together, the results by de Freitas et al. (2018) and Oliveira and Justi (2017a) suggest that morphological awareness predicts single word reading in Brazilian Portuguese at least in the 4th and 5th grades of elementary school (but see Justi & Roazzi, 2012, for an exception). Since some studies have not found evidence for a relationship between morphological awareness and word reading in 2nd and 3rd grades (e.g., Guimarães & Mota, 2016; Miranda & Mota, 2013; Oliveira & Justi, 2017a), it seems that morphological awareness does not predict word reading in Brazilian Portuguese in the early years of elementary school. On the other hand, in a recent study, Oliveira et al. (2020) employed a path analysis and observed that word reading and listening comprehension fully mediated the relationship between morphological awareness and reading comprehension in Brazilian Portuguese-speaking 2nd graders. More importantly, morphological awareness contributed to word reading even when controlling for phonological awareness and nonverbal intelligence. However, one limitation of Oliveira et al. (2020) study is its cross-sectional nature which prevents the authors drawing strong conclusions concerning the direction of the relationship between morphological awareness and single word reading.

The direction of the relationship between morphological awareness and word reading is an important theoretical question (e.g., Deacon et al., 2013; Kruk & Bergman, 2013). Thus, a study that follows children over time and allows for verifying how morphological awareness can predict word reading while controlling for other cognitive abilities, mainly phonological awareness, is necessary. To our knowledge, in the case of Brazilian Portuguese, only one longitudinal study has been reported that evaluated morphological awareness and reading comprehension in 2nd and 3rd graders (Mota et al., 2012). In other words, no longitudinal study has evaluated the relationship between morphological awareness and single word reading in Brazilian Portuguese.

Another issue needing investigation is whether the relationship between morphological awareness and word reading changes with development (e.g., Kirby et al., 2012; Kuo & Anderson, 2006). This is a relevant theoretical question because there are suggestions that the relationship between morphological awareness and reading gets stronger after the initial years of reading instruction (e.g., Kirby et al., 2012; Kuo & Anderson, 2006). It is important to notice that few studies in Brazilian Portuguese (Guimarães & Mota, 2016; Oliveira & Justi, 2017a) considered a broad age range when investigating morphological awareness and word reading. To address this question, we followed children from the 2nd, 3rd, 4th, and 5th grades for 1 year.

## The present study

The present study aims to evaluate longitudinally the relationship between morphological awareness and word reading accuracy in Brazilian children. Thus, children from 2nd to 5th grades were evaluated in the end of the school year and 1 year later. The present study has two main goals: (1) to investigate when morphological awareness contributes to word reading accuracy in Brazilian Portuguese and (2) to investigate longitudinally the direction of this relationship between morphological awareness and word reading in Brazilian Portuguese. Since nonverbal intelligence, phonological awareness, and phonological working memory are well-established predictors of reading that share variance with morphological awareness (e.g., Deacon & Kirby, 2004; Justi & Roazzi, 2012), we included controls for these variables in the present study.

## Theoretical rationale for the present study’s hypotheses

There are different ways by which morphological awareness can contribute to word reading (e.g., Nagy et al, 2014; Levesque et al., 2021). One possibility is morphological decoding (Levesque et al., 2021), that is, using morphological knowledge to decompose a word form in its constituent morphemes to recognize or read aloud a morphological complex word (Levesque et al., 2021). For example, a person can use his or her morphological knowledge to break a word like “react” in its constituent morphemes and use those units to pronounce the word (Deacon et al., 2018). As Deacon et al. (2018) point out, the morphemic boundaries in “reading” and “react” determine the different pronunciations of “ea” in these words.

Considering single word reading in Brazilian Portuguese, we do not expect readers to rely on morphological decoding (Levesque et al., 2021) to sound out words because the grapheme to phoneme mapping in this language is relatively consistent. Thus, we do not see too many opportunities for this kind of strategic morphological decoding (Levesque et al., 2021) to help in the disambiguation of the words pronunciation. For example, think about the Brazilian Portuguese words “refazer” /ʁe.fa.ˈzeʁ/ [TO REMAKE] and “refeitório” /ʁe.fei.ˈtɔ.ɾio/ [REFECTORY]; the “re” in “refazer” is a morpheme; however, the pronounce of the letters “re” /ʁe/ in “refazer” and “refeitório” is the same.

Another possibility for morphological awareness to contribute for word reading is through chunking. A rich structure of morphological knowledge would contribute to tightening the links between the representations of sounds, spellings, and meanings of words and morphemes, supporting the reading and spelling of words in morphemic chunks (Nagy et al., 2014). These chunks would also result in higher quality lexical representations (Perfetti & Hart, 2002) boosting meaning retrieval and word recognition. Thus, as de Freitas et al. (2018) argue, Brazilian Portuguese morphologically complex words such as ‘infelizmente’ [UNHAPPINESS] may be read more quickly if broken into their morphemes “in”- “feliz”- “mente.” In this situation, a person using phonological decoding would achieve the same pronounce; however, the co-activation of the morphemic chunks would result in an easier and more automatic reading.

According to some reading acquisition theories (e.g., Ehri, 2005; Frith, 1985) and visual word recognition models (e.g., Grainger & Beyersman, 2017), the use of morphemic chunks during reading is a later achievement. For example, in Ehri’s (2005) phase theory of reading, it is only in the last phase (the consolidated phase) that grapheme-phoneme connections in the words become consolidated into larger units. According to Ehri (2005), in the consolidated phase, readers retain increasingly more sight words in memory, and, as they become familiar with letter patterns that recur in different words, these letter chunks which include spellings of rimes, syllables, morphemes, and whole words become unitized.

In Grainger and Beyersman’s visual word recognition model (2017), children first establish relationships between whole word orthographic representations and morpho-semantic representations. In this model, it is only with repeated exposure to form meaning relationships that children begin to discover relationships between semantically related morphological units. In addition, affix representations are a later achievement, and they are abstracted away as orthographic units as a consequence of exposure to written words containing the affixes.

As seen above, both in Ehri’s (2005) theory and Grainger and Beyersman’s (2017) model, the use of morphemic chunks during reading would depend on reading experience to abstract away those morphemic chunks in the first place. As a consequence, we do not expect morphological awareness to contribute to word reading in Brazilian Portuguese children in the early years of reading instruction. However, it is important to notice that Ehri’s and Grainger and Beyersman’s theories do not specify age or grade levels for the use of morphemic chunks in their models. Thus, it is not an easy task to establish when it is early or late based only on these models because one phase could start early or late and then extend itself for a longer period depending on reading experience. Considering that in Brazil, the teaching of reading is very unsystematic (Sargiani et al., 2018), and that children are expected to be still automatizing reading in the 3rd grade; it seems reasonable to assume that in 2nd and 3rd graders, reading is not entirely automatized. In addition, in a previous cross-sectional study with children from the 2nd to 5th grades, Oliveira and Justi (2017b) demonstrated that, compared to an orthographic priming condition, Brazilian children exhibited morphological priming effects from the 3rd grade onwards. However, these priming effects occurred in more automatic situations (with a 60 ms stimulus onset ssynchrony) only in the 5th graders. Since in Grainger and Beyersman’s model, morphological priming effects are explained by the co-activation of orthographic representations of shared morphemic chunks, and then based on Oliveira and Justi’s results, it seems fair to assume that it is only in the 5th and maybe 4th grade that Brazilian children can use morphemic chunks during reading.

Once children become more familiar with orthographic patterns corresponding to morphemes, a rich structure of morphological knowledge would contribute to tightening the links between the representations of sounds, spellings, and meanings of words and morphemes (Nagy et al., 2014). Since morphological awareness is also an index of this kind of morphological knowledge (Levesque et al., 2021), we expect a contribution of morphological awareness for word reading after the early years of reading instruction when children are able to use morphemic chunks during reading (from the 4th grade onwards). Furthermore, considering the possibility of a bidirectional relationship between morphological awareness and reading (Deacon et al., 2013), we expect that in the early grades (2nd and 3rd grades), it is word reading that contributes to morphological awareness. This could happen because the exposition to words and word-affix combinations in written language could increase children’s sensitivity to the morphological markings of the words, facilitating the development of morphological abilities (Deacon et al., 2013; Kruk & Bergman, 2013; Kuo & Anderson, 2006).

## Method

The present study performed a longitudinal investigation of the relationship between morphological awareness and reading in 162 children from the 2nd to 5th grades of elementary school. The children were evaluated in the final quarter of the respective school year (time 1) and approximately 1 year later (time 2). The first wave of this study (old cohort) was previously published as cross-sectional data (Oliveira & Justi, 2017a, 2017b) and included additional tasks not used in the present study. The present study added a new sample (new cohort) of children from 2nd to 5th grades to increase sample size and included the retest of all participants approximately 1 year later (second wave for the old cohort and first and second waves for the new cohort). Thus, the present paper presents new and original data relating time 1 to time 2 to evaluate the longitudinal relationship between morphological awareness and reading in Brazilian Portuguese.

### Participants

The full study sample (counting the old and new cohorts) was of 219 children from the 2nd to 5th grades of elementary school. The present study sample consisted only of the 162 children (80 males, and 82 females) who performed all the tasks in time 1 and time 2. The number of children per grade is as follows: forty-two 2nd graders (mean age = 8 years; *SD* = 0.23); forty-three 3rd graders (mean age = 9.02 years; *SD* = 0.40); thirty-eight 4th graders (mean age = 9.93 years; *SD* = 0.35); and thirty-nine 5th graders (mean age = 10.98 years; *SD* = 0.38). The main reasons for the loss of participants from time 1 to time 2 were as follows: children who changed schools, children who missed one of the testing sessions, and children who did not want to participate in time 2. All children had Brazilian Portuguese as their native language and were from two private schools in a city of approximately 500,000 inhabitants in southeastern Brazil.

### Measures

#### Morphological awareness

*Word analogy task* (derivational) (Nunes et al., 1997, adapted by Justi & Roazzi, 2012)

This task is an analogy task with the a:b::c:d format. In this task, first, the experimenter presents a pair of words (a:b) with a morphological relationship (e.g., “ajuda” [HELP] — “ajudante” [HELPER]), and then, a new word (c) is presented to the child (e.g., “jogo” [PLAY]). Using analogical reasoning, the child must respond with a word (d) that completes the second pair (e.g., “jogador” [PLAYER]) presenting the same type of morphological relationship of the first pair. The score on this task equals the number of items answered correctly. It is important to notice that, for a response to be considered correct, it needs to have the same type of morphological relationship of the first pair of words. For example, if the first pair (a:b) was “ajuda”- “ajudante” [HELP-HELPER], and the target word (c) was “jogo” [PLAY], the correct answer (d) is “jogador” [PLAYER]. Thus, if the child merely responds with a semantic-related word which does not present the same type of morphological relationship of the first pair, the answer is considered incorrect (e.g., for the pair “ajuda”- “ajudante” [HELP-HELPER], responding “partida” [MATCH] for the target “jogo” [PLAY] is an incorrect answer). In the present task, we used pairs in which the children could not answer correctly by merely repeating the end sound of the words (e.g., in “ajuda”- “ajudante” and “jogo”- “jogador,” the end sounds are different); thus, to correctly respond in this task, children cannot rely on phonology alone; they need to process the morphological structure of the words. The present task focuses on derivational morphology because only the derivational morphology task presented evidence of validity for discrimination between the 2nd-, 3rd-, and 4th-grade children in the study by Mota et al. (2014) with Brazilian children. The present task has four training items, followed by 12 testing items, and the Cronbach’s alpha of this task is 0.71 (calculated based on the present study’s sample).

#### Word reading

*Reading subtest of the Teste de Desempenho Escolar* [school performance test] (Stein, 1994)

The subtest was applied and scored according to the suggestions of Lucio and Pinheiro (2014), which counts as errors mispronounced words, syllabifications, and self-corrections. Thus, although it is mainly a reading accuracy test, it takes into account bad fluency by penalizing syllabifications and self-corrections. In this test, children need to read aloud a list of 25 words organized in order of difficulty. The majority of the words in this list are morphologically complex (68%). The score on this task equals the number of correctly read aloud words, and according to Lucio and Pineiro (2014), the Cronbach’s alpha for this task is 0.90.

#### Phonological awareness

##### *Phoneme elision task*

Based on the study of Justi and Roazzi (2012), this task involves the oral presentation of a word (e.g., “saco”) and requires the participant to mentally subtract a particular sound (e.g., /s/) and then say the sound that remains (e.g., /aku/). According to Justi and Roazzi (2012), the Cronbach’s alpha of this task is 0.80.

#### Phonological working memory

*Digits subtest of the Wechsler Intelligence Scale for Children* ([WISC-III], Wechsler, 2002)

This subtest was used to evaluate phonological working memory. It consists of two sets of tasks: direct order and reverse order. In the direct order task, the children must repeat a sequence of digits in the same order as that performed by the examiner. In the reverse order task, the children must repeat a sequence of digits in the reverse order as the one performed by the examiner. The subtest was applied and corrected according to the specifications of the manual (Guttman’s split-half reliability of 0.74).

#### Nonverbal intelligence

##### *Raven’s Colored Progressive Matrices test*

This test consists of figures with missing parts that require completion by selecting a correct choice among five choices. The goal is to determine whether children can indicate the part that properly completes the figure. The test was applied and scored according to the Brazilian author’s manual (Angelini, Alves, Custódio, Duarte & Duarte, 1999) and has a Spearman-Brown split-half reliability of 0.92.

### Procedure

Procedures in this study adhere to ethical research policies and were approved by the research ethics committee of the authors’ institution and by the board of the children’s schools. The inclusion criterion was the signing of the informed consent form by the children’s guardians. The exclusion criterion was the presence of uncorrected sensory deficiencies in children, such as blindness or deafness. Oral assent was obtained from each child at every testing session.

The tasks were implemented on days and times agreed upon with the school board and teachers. In time 1 (T1), each child participated individually in two testing sessions of approximately 30 min each. In one session, the Raven’s test was administered, and in the other session, all the other tasks were administered. In time 2 (T2), the children participated only in a session of approximately 30 min, during which the tasks of word reading, morphological awareness, and phonological awareness were administered. All tasks were conducted in a location determined by the respective school board where the research was conducted.

## Results

As there was a loss of participants from time 1 to time 2, one can ask whether the participants who left the study (*N* = 57) performed worse on the word reading test than those who remained in the study (*N* = 162). Thus, we compared the word reading performance of the participants who left the study (*M* = 14.12, *SD* = 6.5), with the word reading performance of the participants who remained in the study (*M* = 13.06, *SD* = 6.6). Since there was no difference between the two groups, *t* (217) = 1.06 and *p* = 0.29, the loss of participants does not seem to have led to a sampling bias. In all the following analyses, we considered data only from the 162 participants who remained in the study (that is, who performed all the tasks in time 1 and time 2).

### Regression analyses

Considering previous research in Brazilian Portuguese (e.g., Coelho & Correa, 2017; de Freitas et al., 2018; Guimarães & Mota, 2016; Miranda & Mota, 2013; Oliveira & Justi, 2017a, 2017b) and the present study’s hypothesis, in the following analyses, the data were divided into two groups: the first group included data concerning T1 2nd and 3rd graders, and the second group included data concerning T1 4th and 5th graders. We adopted this division because, as previously said, in Brazil, reading is expected to be fully automatized at the end of the 3rd grade, and the automatic use of morphemic chunks during reading by Brazilian children appears to be a latter achievement (Oliveira & Justi, 2017b). Once we hypothesized that morphological awareness would not contribute to word reading in the early years of reading instruction, it is reasonable to assume the 2nd and 3rd grades as early years (children are still learning to read) and the 4th and the 5th grades as late years of reading instruction (children are mainly reading to learn). Table 1 presents descriptive statistics for these two groups. Since the skew and kurtosis values for all the variables fell within the acceptable range (Kline, 2005), normality of distribution was assumed for all variables.

**Table 1:** Descriptive statistics of reading, morphological awareness, phonological awareness, phonological working memory, and intelligence tasks

Task	Time 1 grade	*N*	Mean (SD)	Max. T	Skew	Kurtosis
**Time 1**						
Word reading	2nd/3rd	85	9.6 (6.0)	25	0.04	− 1.15
	4th/5th	77	16.9 (4.7)	25	− 0.54	0.06
Morphological awareness	2nd/3rd	85	6.6 (2.7)	12	− 0.38	− 0.47
	4th/5th	77	9.1 (2.0)	12	− 0.86	0.87
Phonological awareness	2nd/3rd	85	6.2 (2.5)	10	− 0.39	− 0.54
	4th/5th	77	7.2 (2.2)	10	− 0.75	− 0.01
Phonological working memory	2nd/3rd	85	10.5 (2.0)	30	0.76	1.47
	4th/5th	77	11.6 (2.5)	30	0.60	− 0.10
Nonverbal IQ	2nd/3rd	85	28.1 (4.3)	36	− 0.40	− 0.35
	4th/5th	77	30.4 (3.3)	36	− 0.97	1.00
**Time 2**						
Word reading	2nd/3rd	85	13.4 (5.3)	25	− 0.12	− 0.70
	4th/5th	77	18.0 (3.8)	25	− 0.11	− 0.82
Morphological awareness	2nd/3rd	85	8.4 (2.3)	12	− 0.35	− 0.50
	4th/5th	77	9.7 (2.1)	12	− 0.87	0.19
Phonological awareness	2nd/3rd	85	7.5 (2.3)	10	− 0.91	0.20
	4th/5th	77	7.7 (2.0)	10	− 1.12	1.85

All scores are raw scores

*SD* Standard deviation, *Max. T* Maximum possible task score

To explore the direction of the relationship between morphological awareness and word reading, multiple linear regression analyses were performed as follows. Word reading and morphological awareness in T2 were alternately used as criteria variables. In all analyses, the Raven, phonological working memory, and phonological awareness tasks were used as controls in step 1. In step 2, the respective autoregressive variable of the criterion variable was considered, and in step 3, the morphological awareness in T1 or word reading in T1 was considered. As pointed out previously, due to the reasonable consistency in the Brazilian Portuguese grapheme-phoneme mapping, we do not expect morphological awareness to predict word reading in the T1 sample of 2nd and 3rd graders (Desrochers et al., 2018; Miranda & Mota, 2013; Oliveira & Justi, 2017a); however, we do expect morphological awareness to predict word reading from the T1 sample of 4th and 5th graders (De Freitas et al., 2018; Oliveira & Justi, 2017a). In addition, considering the possibility of a bidirectional relationship between morphological awareness and reading (Deacon et al., 2013), we hypothesize that word reading will predict morphological awareness in the T1 sample of 2nd and 3rd graders. Table 2 presents the results of the hierarchical regression analyses.

**Table 2:** Hierarchical regression analysis

*T1 grade*	*Criterion*	*Steps*	*Model predictors*	*β*	*R*^*2*^	*ΔR*^*2*^	*Change in F*
2nd/3rd	WR T2	1st	IQ T1 PWM T1 PA T1	0.233** .017 0.325*	0.24	0.24	8.56**
		2nd	WR T1	0.714***	0.62	0.38	81.99**
		3rd	MA T1	.051	0.63	.00	0.48
4th/5th	WR T2	1st	IQ T1 PWM T1 PA T1	0.116 0.184 0.285*	0.21	0.21	6.61**
		2nd	WR 1	0.646***	0.52	0.31	46.65**
		3rd	MA T1	0.282*	0.57	.04	6.99*
2nd/3rd	MA T2	1st	IQ T1 PWM T1 PA T1	0.388*** 0.172 0.157	0.32	0.32	12.6**
		2nd	MA T1	0.252*	0.37	.06	7.0*
		3rd	WR T1	0.324**	0.45	.08	11.4**
4th/5th	MA T2	1st	IQ T1 PWM T1 PA T1	0.131 0.269* 0.214	0.23	0.23	7.3**
		2nd	MA T1	0.747***	0.60	0.37	65.8**
		3rd	WR T1	.011	0.60	.00	.01

*IQ* Nonverbal IQ (Raven), *PWM* Phonological working memory, *PA* Phonological awareness, *MA* Morphological awareness, *WR* Word reading, *T1* Time 1, *T2* Time 2, *Sig.* Significance level of the change in F

*p < .05; **p < .01; ***p < .001

#### Predicting word reading in time 2

As shown in Table 2, the results indicate that in the T1 sample of 2nd and 3rd graders, morphological awareness in time 1 (T1) does not contribute independently to word reading in time 2 (T2) when nonverbal intelligence, phonological working memory, phonological awareness, and word reading in T1 were controlled (*p* = 0.49). Considering all predictors in the model (3rd step), only word reading in T1 was important for predicting word reading in T2 (standardized *β* = 0.71, *p* < 0.01). In the T1 sample of 4th and 5th graders, after controlling for these same variables, T1 morphological awareness contributes to explain an additional 4% of the variation in word reading in T2 (*p* = 0.01). Considering all predictors in the model (3rd step), only word reading in T1 (standardized *β* = 0.53, *p* < 0.01) and morphological awareness in T1 (standardized *β* = 0.28, *p* = 0.01) were important for predicting word reading in T2. The full-standardized *β* for each variable per step can be observed in Table S2 in the supplementary material. In addition, one of the reviewers asked for an analysis of the whole sample with a morphological awareness × grade interaction term. This analysis is presented in Table S1 in the supplemented material. As expected, the interaction term contributed significantly to predicting word reading in T2, which corroborates our division of the sample in early (2nd and 3rd grades) and later (4th and 5th grades) years of reading instruction.

#### Predicting morphological awareness in time 2

The analyses of the T1 sample of 2nd and 3rd graders considering morphological awareness in T2 as a criterion variable showed that T1 word reading contributes to explaining an additional 8% of the morphological awareness variation in T2 (*p* < 0.01) after controlling for nonverbal intelligence, phonological working memory, phonological awareness, and morphological awareness in T1. Considering all predictors in the model (3rd step), nonverbal IQ (standardized *β* = 0.26, *p* = 0.01), word reading in T1 (standardized *β* = 0.32, *p* < 0.01), and morphological awareness in T1 (standardized *β* = 0.24, *p* = 0.01) were important for predicting morphological awareness in T2. In the T1 sample of 4th and 5th graders, after controlling for these same variables, T1 word reading does not contribute to morphological awareness in T2 (*p* = 0.91). Considering all predictors in the model (3rd step), only morphological awareness in T1 was important for predicting morphological awareness in T2 (standardized *β* = 0.74, *p* < 0.01). The full-standardized *β* for each variable per step can be observed in Table S2 in the supplementary material. In addition, one of the reviewers asked for an analysis of the whole sample with a reading × grade interaction term. This analysis is presented in Table S1 in the supplemented material. As expected, the interaction term contributed significantly to predicting morphological awareness in T2, which corroborates our division of the sample in early (2nd and 3rd grades) and later (4th and 5th grades) years of reading instruction.

### Cross-lagged panel correlation analyses

Our hypotheses concerning the relationship between morphological awareness and word reading in early and late years were corroborated. However, to present a more fine-grained analysis of the relationship between morphological awareness and word reading, we decided to follow up the regression analyses with year-by-year cross-lagged panel correlations. Based on the present study hypotheses and previous results, we have reasons to expect that in the T1 sample of 2nd and 3rd graders, it is word reading that influences morphological awareness, and we also have reasons to expect that in the T1 sample of 4th and 5th graders, it is morphological awareness that influences word reading; thus, all the following cross-lagged analyses are one-tailed to increase power. In addition, for every statistically significant difference between the cross-lagged correlations, we also tested for differences between the synchronous correlations, since stationarity is an assumption of the cross-lagged panel correlation (Kenny, 1975). Table 3 presents the Pearson product-moment correlation between morphological awareness and word reading in T1 and T2 for each school grade.

**Table 3:** Person correlations between morphological awareness and word reading across grades

T1 grade	Measures	MA T1	WR T2	MA T2
2nd	WR T1	.054	0.707**	0.456**
	MA T1		0.172	0.421**
	WR T2			0.360*
3rd	WR T1	.047	0.771**	0.394**
	MA T1		.095	0.286
	WR T2			0.423**
4th	WR T1	0.515**	0.676**	0.361*
	MA T1		0.628**	0.751**
	WR T2			0.625**
5th	WR T1	0.659**	0.751**	0.578**
	MA T1		0.569**	0.786**
	WR T2			0.594**

*WR* Word reading, *MA* Morphological awareness, *T1* Time 1, *T2* Time 2

*p < .05; **p < .01

As expected for the early grades, in the T1 sample of 2nd graders, the cross-lags show a statistically significant difference (*Z* =  − 1.72; *p* < 0.05, *one-tailed*; the synchronous correlations were not statistically different, *p* > 0.05) which indicates that word reading influences morphological awareness. The direction of the relationship is the same in the T1 sample of 3rd graders, and the cross-lags show a statistically significant difference (*Z* =  − 1.67; *p* < 0.05, *one-tailed*); however, the synchronous correlations were statistically different (*Z* =  − 2.13, *p* < 0.05). The difference in the synchronous correlations can probably be attributed to an increase in the strength of the relationship between word reading and morphological awareness in time 2 (see Table 3).

Considering the T1 sample of 4th graders, as expected, the relationship changes its direction; the cross-lags show a statistically significant difference (*Z* = 1.98; *p* < 0.05, *one-tailed*; and the synchronous correlations were not statistically different, *p* > 0.05), which indicates that morphological awareness influences word reading. In the T1 sample of 5th graders, the cross-lags did not show a statistically significant difference (*Z* =  − 0.01; *p* > 0.05).

## Discussion

The present study aimed to evaluate the independent contribution of morphological awareness to word reading in Brazilian Portuguese and to investigate the existence of a developmental pattern in this relationship for children from the 2nd to the 5th grades of elementary school. The results of the regression analysis indicate that in the T1 sample of 2nd and 3rd graders, T1 morphological awareness does not contribute independently to word reading 1 year later; however, T1 word reading contributes independently to morphological awareness 1 year later in these same children. On the other hand, considering older children in the present study, we have the opposite pattern, that is, T1 morphological awareness contributes independently to word reading 1 year later, and T1 word reading does not contribute independently to morphological awareness 1 year later in these same children. It is important to notice that the regression analyses were followed up by year-by-year cross-lagged panel correlations. The results of these analyses were virtually the same as those of the regression analysis: word reading influencing morphological awareness in the T1 sample of 2nd and 3rd graders and morphological awareness influencing word reading in the T1 sample of 4th graders. The main difference was in the T1 sample of 5th graders, with a null effect. This lack of statistically significant difference in the 5th grade is probably due to a ceiling effect on morphological awareness measures (mean of 9.62 out of 12 in time 1 and mean of 10.08 in time 2) because the cross-lagged analysis is not appropriate for examining the causal effect of variables that do not change over time (Kenny, 1975). This ceiling effect did not affect the regression analysis results because the data of T1 4th and 5th graders were aggregated in these analyses.

Most Brazilian Portuguese studies that have investigated the relationship between morphological awareness and word reading in children from the 2nd and 3rd grades suggest that the direction of this relationship is from morphological awareness to word reading (e.g., Guimarães, 2005; Guimarães & Mota, 2016; Mota, et al., 2008a, 2008b; Oliveira et al., 2020). However, these studies employed cross-sectional designs, preventing them from drawing reliable inferences concerning the direction of the relationship between the variables. In addition, as pointed out by Oliveira and Justi (2016), few studies that have investigated the relationship between morphological awareness and reading in Brazilian Portuguese have controlled for phonological awareness. Thus, the present study is in better position in this regard because in the regression analysis carried out, even when controlling for nonverbal intelligence, phonological working memory, phonological awareness, and particularly morphological awareness in T1, T1 word reading continued to explain 8% of the morphological awareness variation 1 year later (T2).

One possible explanation for this effect of word reading on morphological awareness in the T1 sample of 2nd and 3rd graders is that reading experience helps children to realize that the spelling of morphologically related words tends to be preserved in Brazilian Portuguese (e.g., “laranja” [ORANGE] and “laranjeira” [ORANGE TREE]). Thus, as they are exposed to a greater number of written words, this enables them to perceive the words’ morphological markings and helps them develop morphological awareness (Deacon et al., 2013).

In relation to the T1 sample of 4th and 5th graders, in the regression analyses, the contribution of T1 morphological awareness to T2 word reading was independent of phonological awareness, phonological working memory, nonverbal intelligence, and initial word reading ability. The cross-lagged panel correlation analyses corroborated these findings except for T1 5th graders (probably due to a ceiling effect on morphological awareness). This result is in line with cross-sectional studies carried out on 4th- and 5th-grade students in Brazil (e.g., de Freitas et al., 2018; Oliveira & Justi, 2017a) and complements these studies, providing longitudinal data indicating that morphological awareness contributes to word reading in these late grades.

As hypothesized, we expected a contribution of morphological awareness for word reading in later grades because morphological awareness can contribute to tightening the links between the representations of sounds, spellings, and meanings of words and morphemes (Nagy et al., 2014; Levesque et al., 2021), resulting in a boost to word recognition due to high-quality lexical representations (Perfetti & Hart, 2002). These high-quality lexical representations would result in an easier and more automatic reading with the co-activation of morphemic chunks. Since these “morpho-orthographic chunks” would depend on letter patterns abstracted away with reading experience (e.g., Ehri, 2005; Grainger & Beyersmann, 2017), it makes sense to think about a later contribution of morphological awareness. After all, the idea of tightening the links between the representations of sounds, spellings, and meanings of words and morphemes (Nagy et al., 2014; Levesque et al., 2021) presupposes that there are orthographic representations of morphemes which could be mapped to sound and meaning.

One question that can be raised is as follows: if morphological awareness depends on some degree of reading experience (abstraction of morpho-othographic chunks) to start contributing to single word reading, how could this happen earlier in opaque languages like English? First, it is important to point out that there are a reasonable number of studies which have not found a morphological awareness contribution for single word reading in English at least until 4th grade (e.g., Apel et al., 2013; Deacon et al., 2004; Nagy et al., 2003). Thus, it is not completely clear how early morphological awareness contributes for single word reading in English. However, assuming that morphological awareness contributes early for word reading in opaque languages, one possible explanation is that in opaque languages like English, children strategically pay attention to letter clusters that represent morphemes and try to sound them out as units, as a way to solve ambiguities which a letter to sound conversion procedure would produce. This idea is in accordance with psycholinguistic grain size theory which assumes that readers of opaque orthographies are likely to rely on larger grain sizes like morphemes, syllables, and rimes when reading aloud (Goswami & Ziegler, 2006; Mousikou et al. 2020; Ziegler & Goswami, 2005). Thus, it is possible that children reading in opaque orthographies become more sensible to the words’ morphology earlier, resulting in a stronger relationship between morphological awareness and single word reading.

On the other hand, in the case of Brazilian Portuguese, and maybe other more transparent orthographies, a simple grapheme to phoneme strategy would be enough for sounding out the words in the early years. Thus, children would have no need to strategically pay attention to larger letter clusters as a way to disambiguate the words pronounce. As a consequence, morphological awareness would not be so important for single word reading in Brazilian Portuguese in these early years. However, morphological awareness would still be important for the development of high-quality lexical representations (Perfetti & Hart, 2002) after some degree of reading experience.

In short, the present study adds novel and important longitudinal data concerning the relationship between morphological awareness and word reading in Brazilian Portuguese, considering a large span of school grades (2nd to 5th grades). It shows that in the early years of reading instruction (T1 sample of 2nd and 3rd graders), it is word reading that contributes to morphological awareness, and that in the more advanced years (T1 sample of 4th and 5th graders), it is morphological awareness which contributes to single word reading. However, it is crucial to notice that these results do not imply a unidirectional relationship between word reading and morphological awareness. They are entirely compatible with a bidirectional interpretation of that relationship (e.g., Deacon et al., 2013; Kruk & Bergman, 2013; Kuo & Anderson, 2006). In this light, it is possible to think that, in the beginning, word reading has a more substantial influence on morphological awareness than morphological awareness has on word reading because experience with written language helps children to realize that the spellings of morphologically related words tend to be preserved, facilitating the perception of the morphological markings of the words and the development of morphological awareness (Deacon et al., 2013). On the other hand, as reading instruction advances and children abstract away more and more “morpho-orthographic chunks” (Ehri, 2005; Grainger & Beyersmann, 2017), the influence of morphological awareness on word reading becomes stronger because morphological awareness helps tighten the links between the representations of sounds, spellings, and meanings of words and morphemes (Nagy et al., 2014; Levesque et al., 2021), resulting in a boost to word recognition due to high-quality lexical representations (Perfetti & Hart, 2002).

### Present study’s limitations

One of the limitations of the present study is the use of only one measure to evaluate morphological awareness. It is certainly better to have more than one measure of a construct; however, the kind of word analogy task used in the present study is the most frequently used task to evaluate morphological awareness in Brazil (Oliveira & Justi, 2016). In addition, as shown in Mota et al., (2008a, 2008b, 2014) studies, the word analogy task (derivational morphology version) is the best morphological awareness task in Brazilian Portuguese considering children from the 2nd to the 4th grades. Thus, we have used probably the best possible task, considering that other morphological awareness tasks easily present ceiling effects in Brazilian Portuguese (see, for example, the flexional morphology task from Mota et al., 2014 or the morphosemantic decision task from Mota et al., (2008a, 2008b) and Oliveira and Justi (2017a).

Another limitation of the present study has to do with the study sample of children from private schools. Considering the city region of the private schools, the present study sample is of children from middle-class families. Thus, one may wonder if these results could generalize to children from lower-income families. In this regard, it is important to notice that the present study replicated the results of the study by de Freitas et al. (2018) conducted with 4th-grade children from public schools (attended mainly by children from poor families). Thus, in both studies, independently of children being from private or public schools, morphological awareness contributed to word reading in 4th graders. In addition, as argued by de Freitas et al. (2018), although socioeconomic status certainly explain variation in metalinguistic and reading skills, there is no clear reason to suspect that the underlying process of learning to read or the relations between metalinguistic skills and reading would differ greatly as a result of socioeconomic status.

One last limitation that we want to discuss is the lack of control of vocabulary. There are reasons to suspect that morphological awareness and vocabulary could influence each other (Kuo & Anderson, 2006; Nagy et al., 2014). However, it is important to notice that a reasonable number of studies had shown that morphological awareness contributes to word reading over and above vocabulary (e.g., Kirby et al., 2012; Deacon et al., 2013; Diamanti et al., 2017; Kruk et al., 2013; Muroya et al., 2017). Thus, instead of controlling for vocabulary, we decided to control for phonological working memory in the present study because phonological working memory could be relevant for performance in the morphological awareness tasks employed in the present study (e.g., Deacon & Kirby, 2004; Justi & Roazzi, 2012). In addition, in comparison with vocabulary, there are not too many studies which have controlled for phonological working memory while investigating the relationship between morphological awareness and single word reading. Just to give an example, none of the above cited studies which controlled for vocabulary controlled for phonological working memory (Deacon et al., 2013; Diamanti et al., 2017; Kirby et al., 2012; Kruk et al., 2013; Muroya et al., 2017). Since practical issues prevent us from controlling for every important variable, we opted to control for phonological working memory because it is already shown that morphological awareness contributes for word reading over and above vocabulary (e.g., Deacon et al., 2013; Diamanti et al., 2017; Kirby et al., 2012; Kruk et al., 2013; Muroya et al., 2017). Thus, depending on how you look at it, this can be viewed as an advantage (controlling for phonological working memory) or as a limitation (lack of control for vocabulary) of the present study.

## Conclusions

The present study adds critical longitudinal data concerning the relationship between morphological awareness and word reading in Brazilian Portuguese, considering a large span of school grades. It shows that in the early years of reading instruction, it is word reading that contributes to morphological awareness, and that in the more advanced years, it is morphological awareness which contributes to word reading. In addition, all these relationships hold even when controlling for intelligence, phonological awareness, and phonological working memory. The present results highlight the importance of conducting reading research in other languages besides English since, in that language, an early contribution of morphological awareness is generally observed.

## Supplementary Information

**Additional file 1: Table S1.** Hierarchical Regression Analyses of the Full Sample with Interaction Terms. **Table S2.** Hierarchical Regression Analysis.

## Data Availability

The datasets used and/or analyzed during the current study are available from the corresponding author on reasonable request.
