# Supplementary material for: The relationship between morphological awareness and word reading in Brazilian Portuguese: a longitudinal study
Source: Psicol Reflex Crit. 2023 Feb 3;36:4. doi: 10.1186/s41155-022-00245-9 (PMC9898478; doi:10.1186/s41155-022-00245-9)
Supplement: Supplementary file 1 — Additional file 1: Table S1. Hierarchical Regression Analyses of the Full Sample with Interaction Terms. Table S2. Hierarchical Regression Analysis. [file 41155_2022_245_MOESM1_ESM.docx]

**Supplementary material**

One of the reviewers of this article suggested an analysis of the full sample with the inclusion of the morphological awareness X grade interaction term. Thus, to explore the direction of the relationship between morphological awareness and word reading, multiple linear regression analyses were performed as follows. Word reading and morphological awareness in T2 were alternately used as criteria variables. In all analyses, the Raven, phonological working memory and phonological awareness tasks were used as controls in step 1. In step 2, either the morphological awareness X grade interaction term or the reading X grade interaction term was included, depending on the criterion variable. Table S1 presents the results of these regression analyses.

Table S1

*Hierarchical Regression Analyses of the Full Sample with Interaction Terms*

| *Criterion* | *Steps* | *Model Predictors* | *Β* | *R^2^* | *ΔR^2^* | *Change in F* |
| --- | --- | --- | --- | --- | --- | --- |
| WR T2 | 1^st^ | IQ T1  PWM T1  PA T1 | .253^***^  .117  .297^**^ | .27 | .27 | 21.20^***^ |
|  | 2^nd^ | MA X GRD T1 | .425^***^ | .40 | .13 | 35.86^***^ |
| MA T2 | 1^st^ | IQ T1  PWM T1  PA T1 | .308^***^  .226^**^  .187^*^ | .32 | .32 | 24.43^***^ |
|  | 2^nd^ | WR X GRD T1 | .297^***^ | .38 | .06 | 16.08^***^ |

*Note*. IQ = Nonverbal IQ (Raven); PWM = Phonological Working Memory; PA = Phonological Awareness; MA X GRD = Morphological Awareness by Grade Interaction Term; WR X GRD = Word Reading by Grade Interaction Term; T1 = Time 1; T2 = Time 2; ^*^ = *p* < .05; ^**^ = *p* < .01; ^***^ = *p* < .001.

Table S2

*Hierarchical Regression Analysis*

| *T1 Grade* | *Criterion* | *Steps* | *Model Predictors* | *β* | *R^2^* | *ΔR^2^* | *Change in F* |
| --- | --- | --- | --- | --- | --- | --- | --- |
| 2^nd^/3^rd^ | WR T2 | 1^st^ | IQ T1  PWM T1  PA T1 | .233^**^  .017  .325^*^ | .24 | .24 | 8.56^**^ |
|  |  | 2^nd^ | IQ T1  PWM T1  PA T1  WR T1 | .044  .037  .098  .714^***^ | .62 | .38 | 81.99^**^ |
|  |  | 3^rd^ | IQ T1  PWM T1  PA T1  WR T1  MA T1 | .037  .027  .093  .712  .051 | .63 | .00 | .48 |
| 4^th^/5^th^ | WR T2 | 1^st^ | IQ T1  PWM T1  PA T1 | .116  .184  .285^*^ | .21 | .21 | 6.61^**^ |
|  |  | 2^nd^ | IQ T1  PWM T1  PA T1  WR 1 | .000  .126  .052  .646^***^ | .52 | .31 | 46.65^**^ |
|  |  | 3^rd^ | IQ T1  PWM T1  PA T1  WR 1  MA T1 | -.025  .038  .027  .533^***^  .282^*^ | .57 | .04 | 6.99^*^ |
| 2^nd^/3^rd^ | MA T2 | 1^st^ | IQ T1  PWM T1  PA T1 | .388^***^  .172  .157 | .32 | .32 | 12.6^**^ |
|  |  | 2^nd^ | IQ T1  PWM T1  PA T1  MA T1 | .350^**^  .119  .128  .252^*^ | .37 | .06 | 7.0^*^ |
|  |  | 3^rd^ | IQ T1  PWM T1  PA T1  MA T1  WR T1 | .265^*^  .130  .025  .244^**^  .324^**^ | .45 | .08 | 11.4^**^ |
| 4^th^/5^th^ | MA T2 | 1^st^ | IQ T1  PWM T1  PA T1 | .131  .269^*^  .214 | .23 | .23 | 7.3^**^ |
|  |  | 2^nd^ | IQ T1  PWM T1  PA T1  MA T1 | .009  .010  .038  .747^***^ | .60 | .37 | 65.8^**^ |
|  |  | 3^rd^ | IQ T1  PWM T1  PA T1  MA T1  WR T1 | .008  .010  .035  .742  .011 | .60 | .00 | .01 |

*Note*. IQ = Nonverbal IQ (Raven); PWM = Phonological Working Memory; PA = Phonological Awareness; MA = Morphological Awareness; WR = Word Reading; T1 = Time 1; T2 = Time 2; Sig. = significance level of the change in F; ^*^ = *p* < .05; ^**^ = *p* < .01; ^***^ = *p* < .001.
